# Antimicrobial Potential of Single Metabolites of Curcuma longa Assessed in the Total Extract by Thin-Layer Chromatography-Based Bioautography and Image Analysis

**DOI:** 10.3390/ijms20040898

**Published:** 2019-02-19

**Authors:** Lidia Czernicka, Agnieszka Grzegorczyk, Zbigniew Marzec, Beata Antosiewicz, Anna Malm, Wirginia Kukula-Koch

**Affiliations:** 1 Chair and Department of Food and Nutrition, Medical University of Lublin, 4a Chodźki Str., 20-093 Lublin, Poland; lidiaczernicka@umlub.pl (L.C.); zbigniew.marzec@umlub.pl (Z.M.); 2 Department of Pharmaceutical Microbiology with Laboratory for Microbiological Diagnostics, Faculty of Pharmacy with Medical Analytics Division, Medical University of Lublin, 1 Chodźki, Lublin 20-093, Poland; agnieszka.grzegorczyk@umlub.pl (A.G.); anna.malm@umlub.pl (A.M.); 3 Department of Cosmetology, University of Information Technology and Management in Rzeszow, 35-225 Rzeszów, Poland; bantosiewicz@wsiz.rzeszow.pl; 4 Chair and Department of Pharmacognosy with Medicinal Plants Unit, Medical University of Lublin, 20-093 Lublin, Poland

**Keywords:** *Curcuma longa*, turmeric tuber, Zingiberaceae, TLC bioautography, antimicrobial agents, ImageJ, TLC-MS, hydrostatic counter-current chromatography, centrifugal partition chromatography

## Abstract

*Curcuma longa* from Zingiberaceae belongs to the major spices consumed around the world, known from its cholagogue, anti-inflammatory, and antimicrobial properties. Lack of data on the activity of single components of turmeric extract encouraged the authors to apply TLC (thin-layer chromatography) based bioautography studies to reveal its antimicrobial constituents and construct a universal platform for the bioactivity assessment of crude extracts, with help of a freeware ImageJ software. This optimized chromatographic bioassay performed on diethyl ether and methanol extracts of *Curcuma longa* was successfully applied on the total extract and revealed the antimicrobial potential of single components against a variety of Gram-positive strains, with no need for their isolation from the mixture. The obtained results were further confronted with a classic microdilution antimicrobial assay on the isolates, purified from the crude extracts by centrifugal partition chromatography in the following solvent system: heptane-chloroform-methanol-water (5:6:3:2) (*v*/*v*/*v*/*v*).

## 1. Introduction

In recent years, the development of bacterial resistance against antibiotics available in the pharmaceutical market constitutes a serious pharmacological issue. There are many cases of hospital infections, bacterial diseases originating from tropical countries, or infections caused by mutant bacteria, which are difficult to treat [1]. Since the beginning of the 80 s, there have been numerous attempts to discover new potent antimicrobial agents and develop novel antibiotics that can overcome severe problems associated with bacterial resistance. Even though the costs of conducted studies are high, as in any field of research, the results still do not bring much novelty to the field. Moreover, significant adverse or side effects caused by the currently administered drugs to the macroorganism constitute another important issue that needs a special focus. For these reasons, phytotherapy has become a popular field of study for both patients searching for new and more effective therapeutical strategies and for researchers searching for ideas in the process of new drug design, as nature has already given a variety of chemical structures to modern pharmacology.

Turmeric (*Curcuma longa* L.) is one of the most widely recognized medicinal plants. It is a tropical, perennial spice that belongs to the Zingiberaceae family and is widely cultivated in Malaysia, India, and Indonesia for its flavoring, coloring, and medicinal applications [2], together with its low toxicity (doses of 12 g/day are reported to be safe to humans [3]). The first information about its medicinal uses come from Ayurveda, Siddha, and Unani medicine systems and concerns its antibacterial, wound healing, anti-inflammatory, digestive, and anticancer properties [4]. 

Turmeric extracts contain both precious sesquiterpenes (α-zingiberene, β-sesquiphellandrene, and *ar*-turmerone as the leading constituents of terpene fraction) and polyphenols (curcuminoids). Among the latter group, curcumin, demethoxycurcumin, and bisdemethoxycurcumin can be distinguished as the leading compounds [2]. These ferulic acid derivatives are responsible for the major pharmacological effects of turmeric. Their strong antioxidant potential affects the inhibition of tumor growth and promotes antisclerotic or neuroprotective activity of the plant [5]. Interestingly, the majority of scientific publications describe the activity of crude extracts or focus on the properties of curcumin—the major phenolic metabolite of the plant. Because of some difficulties in the isolation of curcuminoids caused by a marked structural similarity between one another, much less is known about the potential of the remaining constituents of this plant. 

Several publications have reported the antimicrobial activity of turmeric extract against oral bacteria [6,7] or compared its potential by considering the extraction conditions used [8,9,10]. A large majority of the papers, however, describe the antibacterial potential of the crude turmeric extract or crude turmeric oil [11,12,13,14].

The aim of this study was to evaluate the antimicrobial potential of single constituents of methanol and diethyl ether extracts of *C. longa* L., without any need for their isolation from the crude extract. This task was performed by the application of TLC bioautography with image analysis on the selected strains of Gram-positive bacteria, and the result was expressed as the antimicrobial potential according to the values of minimum inhibitory quantity (MIQ) of the compounds. The subsequent identification of potent components was performed using a TLC-MS interface in a direct MS analysis of an indicated spot, which clearly determined the structure of the analyzed compounds. The accuracy of this methodology was assessed by the authors on the isolated single metabolites in the microdilution test in order to compare the results with those from image analyses. The latter task was performed through the application of centrifugal partition chromatography (CPC) on the crude extract.

This study was performed to show a new approach—the performance of a rapid screening protocol that enables the identification of natural antimicrobial metabolites present in the mixtures and can be applied in the studies of other plant extracts.

## 2. Results and Discussion

Antimicrobial potential of the members of the Zingiberaceae family has been extensively studied in recent years. Turmeric, one of the most known species of this family, is perceived as an efficient antimicrobial herb with low toxicity, which can be supplemented or eaten as a spice [15]. The authors in their works underline a marked potential of both—essential oil and alcoholic extracts obtained from different species of *Curcuma* grown worldwide. Although there are many reports on the activity of the total extracts, only a few papers have focused on the potential of single metabolites present in turmeric tuber [16,17,18]. None of them have actually answered the question on which metabolites plays a crucial role in the process of bacterial growth inhibition. 

The present work shows the efficiency of chromatographic separation coupled with image processing software in the estimation of antimicrobial activity of turmeric metabolites, tested against a large selection of Gram-positive bacteria. 

### 2.1. LC-ESI-Q-TOF-MS Analysis of the Extracts’ Qualitative and Quantitative Composition

Qualitative and quantitative composition of the obtained extracts was studied by a mass spectrometer coupled with liquid chromatography, which provided high accuracy mass measurements with low error of measurement values. Several compounds were identified by the authors in both extracts on the basis of the fragmentation patterns and the available scientific literature. For the purpose of this study, the below-described inhibitors of bacterial growth were quantified. 

The optimized methodology allowed high sensitivity of the measurement and precise determination of molecular formulas, without any doubt concerning the number of defined elements in the metabolites’ structures. The positive ionization mode was found to be preferable for the determination of both phenolic compounds and terpenes present in the turmeric. The applied fragmentation voltage of 150 V and the CID energies of 10 and 20 V allowed sufficient sensitivity and good fragmentation of molecular ions.

Table 1 shows the list of identified components of the evaluated turmeric extracts together with the recognition of other major peaks present in the analyzed sample with the total ion chromatogram and their spectral data. All identified compounds were found in both MeOH and diethyl ether extracts. The extracted ion chromatograms of the described components are present in the Supplementary file.

The first four identified components are the compounds later confirmed to be responsible for the antimicrobial activity. They constitute 22.1 ± 0.4% (CU), 9.14 ± 0.2% (DMCU), 11.33 ± 0.2% (BDMCU), and 3.48 ± 0.06% (TUR) of the MeOH extract and 19.2 ± 0.2% (CU), 7.21 ± 0.4% (DMCU), 8.95 ± 0.3% (BDMCU), and 4.58 ± 0.1% (TUR) of the diethyl ether extract of turmeric tubers, measured in four separate injections of the extracts. As presented above, the influence of the extractant used on the final composition of the extracts was not significant.

Our studies on the composition of the extracts are in accordance with the previously published papers. Other authors describe a similar ratio of these three pigments with curcumin as the leading component, however, the total concentration of those differ depending on the place of origin. According to the studies of Peret-Almeida and co-workers (2008) the quantity of curcumin reached 50%, DMCU—29% and BDMCU—12% in the extract. According to the studies of Jayaprakasha and colleagues, the ferulic acid derivatives varied in four varieties of the plant and stayed within the range of 1.06–5.65%, DMCU: 0.83–3.36% and BDMCU: 0.42–2.16% in the tubers [19,20].

Detailed LC-MS analysis of the extract’ composition revealed the presence of other phenolic compounds, which were tentatively identified by the authors based on the scientific literature and fragmentation patterns [21]. The derivatives of the investigated three pigments are characterized by a different quantity of unsaturated bonds. Even if present in the extracts, their concentration was scarce (Table 1).

### 2.2. TLC Bioautography Tests and Identification of Active Metabolites by TLC-LC-ESI-TOF-MS

From the optimization studies, two mobile phases were selected for the analyses of extracts: MeOH: DCM (0.5: 9.5 *v*/*v*) for the more polar MeOH extract, and n-hexane: DCM (1:9 *v*/*v*) for the diethyl ether extract. The evaluated conditions provided effective separation of both polar and nonpolar compounds present in the extracts. 

As mentioned in the Materials and Methods section, seven strains of Gram-positive bacteria were used for the determination of antimicrobial properties of turmeric. All the strains grew well on the MHA agar, and after re-incubation with TTC, they provided evenly colored plates with colorless zones of inhibition. The preliminary studies revealed that the most efficient bacterial inoculum concentration was 1.5 × 10^6^ CFU; it assured an even colorization of the plate with no zones of color lightening. An absolute condition for conducting the tests was the need to dry out the mobile phase from the TLC plates before applying the bacteria strain and to keep them closed inside the Petri dishes prior to covering with the medium.

In the next step of the experiment, on the uncoated but developed TLC plates, all zones corresponding to the zones of inhibition were analyzed by a mass spectrometer. In the optimized TLC development conditions for the identification of single components present on the uncoated TLC plates were afforded by a TLC-MS interface, thus providing high accuracy mass measurements of metabolites and their fragmentation patterns; this allowed data- and literature-based identification [15,21]. Among the metabolites which were found to be active in the applied concentrations of the total extracts, three curcuminoids—curcumin (CU), demethoxycurcumin (DMCU), and bisdemethoxycurcumin (BDMCU)—in both extracts and turmerone (TUR) in the nonpolar extract were identified. Table S1 (in the Supplementary file) presents the identity of these metabolites, the total chromatograms of the analyzed spots, and the MS spectra of major peaks. All the below-presented data were obtained in the positive ionization mode.

The behavior of the identified metabolites was different, depending on the strain tested. Table 2. shows the bacterial strains sensitive to the identified compounds. The obtained values come from a careful analysis of all developed TLC plates. The spots of the lowest extract concentration, with an unambiguous red color clearance were selected for the determination of MIQ value. To obtain the final result, the concentration of the spotted total extract was divided by the actual content of each curcuminoid and *ar*-turmerone in the extract to deliver the values presented in Table 2, which constitute an estimated quantification.

Differences in the action of individual compounds result from the varied sensitivity of each microorganism and each strain of a given species. Typically, *S. epidermidis* or *B. subtilis* are more sensitive than *S. aureus*. In addition, the *S. aureus* ATCC 43300 strain is a methicillin resistant strain, and therefore may be less susceptible to tested drugs.

*ar-*Turmerone did not inhibit all tested bacteria. It was found to be active against *S. aureus* ATCC 43300, *S. epidermidis* ATCC 12228, and *B. subtilis* ATCC 6633, with the lowest MIQ value for the first strain.

As previously mentioned, the studies on the antimicrobial potential of turmeric metabolites are scarce. The activity of ar-turmerone described in this paper is, however, in accordance with the publication of Peret-Almeida and colleagues [19], who confirmed the antimicrobial activity of turmeric oil with the main metabolite identified as ar-turmerone, against *Bacillus subtilis* (the diameters around the discs increased by 131%) [15]. 

Interestingly, all curcuminoids inhibited the growth of bacteria. Considering their concentration in the total extract, as described above, BDMCU, as a less abundant component in the extract, showed the strongest inhibition of the growth of bacteria. Its lowest MIQ values were noted for *S. aureus* ATCC 43300 (MIQ = 0.28 µg) and *B. cereus* ATCC 10876 (MIQ ≤ 0.14 µg)), whereas its weakest activity was observed for two strains: *S. aureus* ATCC 25923 and *S. aureus* ATCC 43300. In the only study on single curcuminoids antimicrobial activity which is known to the authors, BDMCU was also found the most active among the tested three curcuminoids, with a particular antimicrobial potential against *Bacillus subtilis* at a concentration of 10 µg/µL [15].

The other curcumin derivative—DMCU—even if present in the smallest quantity, was found to show stronger inhibitory properties from curcumin itself. *Bacillus cereus* ATCC 10876 was the most susceptible to DMCU (MIQ = <0.11 µg) among all four studied compounds.

Interestingly, similar tendency in the pharmacological activity strength of the herein described phenolics was also observed in the works related to the anticancer activity assessment [22]. 

### 2.3. Purification of Single Curcuminoids by CPC Chromatography

The applied separation conditions enabled a successful purification of curcuminoids: bisdemethoxycurcumin was present in fractions no. 8–12, demethoxycurcumin in fractions no. 16–18, and curcumin in fractions no. 22–26. The identification of the ferulic acid derivatives and their purity was performed by LC-MS spectrometry on the basis of their fragmentation pattern, the retention time, and the scientific literature, as shown in the Supplementary Material (Figures S2 and S3). The purity of all compounds exceeded 95.5%, and for curcumin, the purity was calculated as 98.2%. From the injected total extract, the authors obtained as much as 178 mg of curcumin, which was used to perform a microdilution antimicrobial assay. The data on the purity of isolated curcumin are presented in the Supplementary file (Figure S1).

A standard microdilution antimicrobial assay was performed on the sample of pure curcumin to assess the accuracy of the TLC bioautography technique.

### 2.4. Antibacterial Potential of Turmeric Metabolites in Relation to the Commonly-Used Antibiotic Vancomycin

As shown in the Table 2 and Table 3, the MIQ values obtained for curcumin differ, depending on the method used. This result seems to be valid as the quantity of microorganisms in both tests differs significantly. The concept of microdilution assay is to study a sample dipped in the medium containing a certain concentration of bacteria. As a consequence, the tested sample can interact with all bacterial cells inside the well. In the TLC bioautography tests, the effect of tested compounds on the microorganisms is limited to the place where the spot is located; thus, every metabolite affects a much smaller quantity of the microorganisms than in the microdilution assay. 

To relate the bioautographic results presented in Table 2 to those obtained by the standard microdilution assay, we found it necessary to analyze the behavior of the reference compound vancomycin on a TLC plate under the same analytical conditions as those used for the extracts.

For this purpose, chromatograms with different concentrations of vancomycin were prepared. During the chromatogram processing of images, the concentration of the antibacterial activity of the compounds was reflected in their peak areas. The higher the concentration, the larger the absolute value of its peak area. 

The set of peak areas of vancomycin obtained from an ImageJ image processing was used for the preparation of calibration curves for each bacterial strain evaluated in the study.

The calibration curve equations are presented in Table 4. All of them showed high linearity (R^2^ values were higher than 0.95) and could be successfully used for the determination of vancomycin equivalents, which characterize the tested natural products. Table 4 shows the calculations of vancomycin equivalents for curcumin, as the major constituent of the extracts, determined in both antimicrobial assays.

The above-presented data show that the findings of TLC-based bioautographic test were identical to the results of antimicrobial studies performed in a traditional way by using the microdilution method and were performed by the authors of the manuscript [23]. With a relatively low error, the antimicrobial potential of curcumin could be assessed without any need for their purification from the total extract. 

The herein presented approach is universal and may be applied to the analyses of other complex matrices such as plant extracts, where the process of isolation is time-, money- and solvent-consuming.

Image processing by the freeware software ImageJ program is an interesting alternative for biological screening studies, as it is easy to operate and offers a wide range of image modifications. Initial screening of biological properties by imaging software may provide important information on the pharmacological potential of the ingredients of various mixtures, without any need for their isolation; this favors the green chemistry approach and reduces the efforts of researchers. 

## 3. Materials and Methods

### 3.1. General

Silica gel 60 F254 neutral TLC plates were purchased from Merck (Darmstadt, Germany) for TLC bioautography assays. Hamilton micropipette 705 (Hamilton, Bonaduz, Switzerland) was used for the extracts’ application onto the TLC plates. TLC chromatograms were photographed with a Canon Power Shot G5 camera digital camera with standard 18-35 lens. The pictures were processed using an ImageJ 1.48v image processing program (Wayne Rasband, National Institutes of Health, Maryland, USA).

An accelerated solvent extraction apparatus (ASE, Model ASE 100, Dionex, CA, USA) was used for the extraction of plant material. A centrifugal partition chromatograph SCPC-250-L system by Armen (Saint Ave, France) equipped with a 250 mL column, a quaternary pump, a fraction collector (LS-5600) and a UV detector (Flesh06S DAD 600) provided the isolation of curcuminoids.

An LC-ESI-Q-TOF-MS qualitative and quantitative analysis of extracts and isolates was performed using an Agilent G3250AA LC/MSD TOF system containing a photodiode array detector (G1315D), an HP 1200 chromatograph and a Q-TOF-MS spectrometer (Agilent Technologies, Santa Clara, CA, USA) with an ESI ionization source. The apparatus contained a column thermostate, a degasser (G1322A), an autosampler (G1329B) and the binary pump (G1312C). The analysis was performed on an HPLC column: Zorbax RP 18 (150 mm × 2.1 mm, dp = 3.5 µm) with a fast gradient elution method. 

The identification of active components directly from a TLC plate was performed using an LC-ESI-TOF spectrometer Agilent Technologies (Santa Clara, CA, USA) equipped with an HPLC system G3250AA LC composed of a binary pump, an autosampler, a thermostated column, a photodiode-array (PDA) detector and a degasser, and a mass spectrometer: 6210 MSD TOF with ESI dual-spray source. The system with a TLC-MS interface (Camag, Muttenz, Switzerland) contained a Zorbax RP-18 Rapid Resolution column (50 × 2.1 mm, 5 µm diameter).

### 3.2. Chemicals and Reagents

The chemicals used in the study: methanol, ethanol, dichloromethane, diethyl ether, n-hexane, ammonia, sulphuric acid, heptane, chloroform, and vanillin were produced by Avantor Performance Materials (Gliwice, Poland), whereas water and acetonitrile of spectroscopic grade were obtained from Merck (Darmstadt, Germany). Vancomycin was purchased from Sigma Aldrich (St. Louis, CA, USA) as well as 2,3,5-triphenyltetrazolium chloride (TTC), which is the dye used in this work to observe, stain colonies of microorganisms on solid culture media such as Mueller-Hinton Agar (MHA) on TLC plates.

### 3.3. Plant Extract Preparation

Fresh turmeric rhizomes were purchased from Tex Plants & More Philipp Foerster, Annaberg-Buchholz, Germany; they were immediately frozen after the delivery and then thawed in the required quantity shortly before the extraction. For the preparation of extracts, the tubers were cut into small pieces and placed in a 30 mL stainless steel extraction cell with a cellulose filter at the bottom end (methanol extracts) or in a mortar (diethyl ether extracts). Two types of extractants were used to enrich the final extract either in more or less polar constituents to obtain a more comprehensive picture in the antimicrobial tests. The extraction with methanol was conducted under the following conditions in an ASE apparatus: temperature, 70 °C; purge volume, 70%; purge time, 100 s; analysis time, 10 min; number of cycles, 3; this extraction was done after an initial purification from unipolar components with n-hexane under the same conditions, but with 1 cycle [22]. The extraction with diethyl ether was performed by pounding crushed turmeric rhizomes in a mortar with solvent, followed by drying of the sample over silica gel. Methanol extracts were evaporated on a rotary evaporator at the temperature of 45 °C and re-dissolved in methanol to obtain following concentrations: 2, 1, 0.5, 0.25, 0.125, and 0.0625 mg/mL. Diethyl ether extracts were left under the fume hood until they became dry. Methanol was used to obtain similar concentrations as those for the other extracts.

### 3.4. Thin-Layer Chromatography (TLC) Conditions

TLC chromatograms were performed using aluminum TLC plates (silica gel 60 F254, Merck, Darmstadt, Germany). 

An initial evaluation of the chromatographic conditions and the composition of a solvent system was performed using a 1% solution of vanillin, which provided a successful visualization of the extracts’ constituents. The 1% vanillin-sulfuric acid reagent was prepared by dissolving 2 mL of sulfuric acid in a 1% methanolic solution of vanillin (*w*/*v*). After spraying, the TLC plates were heated at 110 °C for optimal color development. 

Every plate used in the TLC bioautography studies was sized 7 × 6 cm and contained six concentrations of each extract, namely 2, 1, 0.5, 0.25, 0.125, and 0.0625 mg/mL, spotted quantitatively by a Hamilton syringe with the following volumes, respectively: 20, 10, 5, 2.5, 1.25, and 0.675 µL. The applied volume resembled 40, 20, 10, 5, 2.5, and 1.25 µg of extract within the spot. Thus, 16 TLC plates were prepared for both extracts for each tested bacterial strain. 

After the color development, the TLC plates were dried inside sterile Petri dishes in a laminar flow hood to minimize microbial contamination from the air. The active compounds were then visualized under UV light at 254 nm and 366 nm and marked on each TLC plate with a pencil. After drying they were subjected to further microbiological analysis. Each TLC plate was prepared in triplicate. Each time, three TLC plates were coated with a bacterial broth. Additionally, six TLC plates for each extract were prepared for the identification and quantitative studies. 

### 3.5. Organisms and Media

Several Gram-positive bacterial strains applied onto the developed TLC plates for the estimation of active metabolites of both extracts: *Staphylococcus aureus* ATCC 25923, *Staphylococcus aureus* ATCC 6538, *Staphylococcus aureus* ATCC 43300, *Staphylococcus epidermidis* ATCC 12228, *Bacillus subtilis* ATCC 6633, *Bacillus cereus* ATCC 10876, and *Micrococcus luteus* ATCC 10240. The above microorganisms were obtained from American Type Culture Collection, which are used as microbiological standards in the study of the activity of various substances and compounds against microbes.

### 3.6. Bioautographic Methods

The developed TLC plates were covered (sprayed) manually by MHA containing the bacterial inoculum of 1.5 × 10^6^ CFU (colony forming units)/mL. These bioautograms were placed in sterile Petri dishes and were incubated at 35 °C for 24 h under aerobic and humid conditions. After the completion of incubation, TTC was used to visualize zones of inhibition of bacterial growth. These salts were sprayed onto the bioautograms and were reincubated at 35 °C for 24 h. Transparent zones of bacterial growth inhibition indicated the antibacterial activity of the samples.

Additionally, vancomycin—an antibiotic with a wide spectrum of antibacterial activity against Gram-positive bacteria—was used to define the sensitivity of the assay. Several concentrations of the antibiotic were prepared as follows: 128, 32, 8, 2, 0.5, and 0.2 µg/mL (for *Staphylococcus aureus* ATCC 6538), and 64, 16, 4, 1, 0.25, and 0.1 µg/mL (for *S. aureus* ATCC 25923, *Bacillus subtilis* ATCC 6633, *Micrococcus luteus* ATCC 10240, *S. aureus* ATCC 43300, and *Bacillus cereus* ATCC 10876), and the solutions were spotted on the TLC plates in the following volumes: 20, 10, 5, 2.5, 1.25, and 0.675 µL, respectively. The test concentrations of this antibiotic were selected on the basis of previous studies, where this compound was studied in the microdilution tests, [24,25], and on the basis of the values of peak areas of active components of the extracts for the calibration curves to stay within a similar range.

#### Determination of the MIQ Values

MIQ values are the lowest concentration of a chemical compound that inhibits the visible growth of a microorganism after incubation [26]. In the present bioautography assay, the MIC values were determined from a series of dilution of samples, similar to a classical microdilution assay. The inhibition zones were observed as yellowish or clear areas against a red background on the plates. The lowest quantity of a compound that inhibited the growth of bacterial strain was considered as the MIQ value. On the basis of the obtained peak areas and known concentrations of active components in the total extract, the MIQ values were calculated for each active compound and each strain. Furthermore, to express the antimicrobial potential of metabolites relative to known reference standards, their antimicrobial potential was expressed in vancomycin equivalents, in respect to the prepared calibration curve equations for each bacterial strain. 

To confirm the accuracy of TLC bioautography-based methodology the antimicrobial potential was also assessed for one of the active antimicrobial metabolites—curcumin, which was isolated by the authors by using centrifugal partition chromatography (see Table 4) and tested by the microdilution method according to the previously described methodology [27].

### 3.7. Image Processing

ImageJ—an image processing program—was used for the measurement of the area of inhibition zones to define the antimicrobial potential of single metabolites in the total extract (to determine their MIQ values and vancomycin equivalents) [28]. 

After 24 hours of incubation of TLC plates with the bacterial strain, the developed TLC plates were photographed by Canon Power Shot G5 camera. The obtained photos in the JPG format were processed by free, open source image processing software—ImageJ program 1.48 v—to quantitatively determine the MIQ values of all active constituents. 

**Figure 1:**
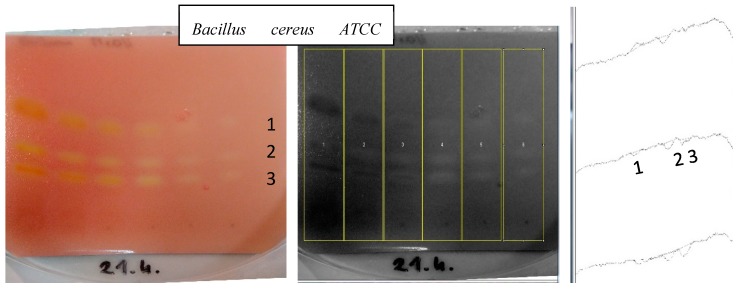
Determination of peak areas of active metabolites visualized by TLC bioautography. The example shows the TLC plate with MeOH extract of turmeric, covered with *B. cereus* ATCC 10876 strain, which was developed on NP silica gel TLC plates by using MeOH: DCM (0.5:99.5 *v*/*v*) as the mobile phase. The picture on the right shows the obtained densitometric data from the program.

First, all documented photos were modified according to the procedure described by Olech et al. [20]. The images were converted into 8-bit type pictures, as initially they contained white or yellowish spots of active components against a red background, which would interfere with proper area quantitation. After this transformation, the spots were read as white against a dark gray background. Later the median filter was set at 5 pixels and the FFT Bandpass filter for large structures—was moved down to 40 pixels. To prepare an outline of the track, a “rectangular selection tool” parameter was selected, and the profile lines were then generated with the “plot lines” option. Thus, videoscan images were converted into chromatograms. Then, a “straight line selection tool” was applied to draw the baseline and the “wand tool” was used to determine the peak areas. The peaks of active metabolites were recorded as negative signals to the track’s baseline (see Figure 1.). 

### 3.8. Quantitative and Qualitative Analysis of the Samples by Mass Spectrometry

All obtained extracts and purified compounds were analyzed by the LC-ESI-Q-TOF-MS spectrometer from Agilent Technologies (see Section 2.1) in both positive and negative ionization modes. The analyses were performed on a Zorbax RP 18 (150 × 2.1 mm, d_p_ = 3.5 µm) chromatographic column using the following gradient of acetonitrile with 0.1% formic acid (FA) (solvent B) and 0.1% aqueous FA (solvent A): t = 0 min: 30% B, t = 20 min: 45% B, t = 20.1 min: 70% B, and t = 30 min: 60% B. The injection volume was 10 µL per sample, the flow rate: 0.2 mL/min and the post run was set at 5 min. Other operation parameters are listed below: drying gas flow: 12 L/min, nebulizer voltage: 35 psi, fragmentor voltage: 150 V, skimmer voltage: 65 V, capillary and vaporizer temperatures: 350 °C each, capillary voltage: 3.5 kV, the wavelength range of PDA detector: 190–500 nm. All spectra were recorded in both ionization modes in the mass range of 40–1000 m/z. The MS/MS spectra were automatically recorded for two the most intensive *m/z* signals in two CID energies: 10 and 20 V, which were later excluded for the following 0.3 min from fragmentation. The identification of metabolites was based on the retention times, fragmentation patterns, and scientific literature.

Additionally, a TLC-LC-ESI-TOF-MS (see Section 2.1) analysis was conducted on the developed TLC plates without derivatization (one TLC for each extract at different concentrations) to identify the spots responsible for the antimicrobial activity in the tested extracts and to check their purity. For the sake of quantitative estimations, the peak areas of the eventually occurring impurities were later subtracted from the total peak area of each active spot. That is why, the chromatograms were obtained in triplicate.

For this purpose, a simple gradient method was applied on a short chromatographic column. The spots of interest not covered with medium and/or bacteria, were collected by a TLC-MS interface (Camag, Muttenz, Switzerland) and the identification of each spot was performed by analyzing their MS spectra. The impurities were not visible in the chromatograms thanks to the mounting of a short chromatographic column (see Section 2.1) before the detectors. The following conditions of LC-MS operation were applied: gas temperature—350 °C, gas flow—10 L/min, *m/z* range—100–1500, nebulizer pressure—30 psi, capillary voltage—3500 V, fragmentor voltage—150 V, skimmer—65 V. The applied gradient of solvents (A—0.1% formic acid, and B—0.1% of formic acid in acetonitrile) was composed of the following steps: 0 min—35% B, 2 min—95% B, 3 min—95% B, 3.1 min—35% B. The run length was set at 10 min, the flow rate at 0.2 mL/ min, and the injection time at 3 sec.

### 3.9. Application of CPC Chromatography in the Isolation of Active Components of Turmeric Extract

To assess the accuracy of TLC-based bioautographic antimicrobial tests, the authors used previously reported separation conditions [22] to obtain pure curcumin and study its antimicrobial potential in a standard microdilution antimicrobial assay. These results were compared with those theoretically calculated using the imaging program.

Curcumin purification was performed using a centrifugal partition chromatograph SCPC-250-L from Armen (Saint-Ave, France), according to the method previously published by the authors [15]. The biphasic solvent system used in the separation process contained a mixture of heptane-chloroform-methanol-water (5:6:3:2) (*v*/*v*/*v*/*v*) and was prepared according to the calculations of partition coefficient values of all major peaks present in the chromatograms of the total extract. The following experimental conditions were applied: rotation speed: 1200 rpm, UV detection: 425 nm and 290 nm, flow rate: 6 mL/min, injection volume: 6 mL, mode of operation: ascending. The purification of active metabolites from turmeric was performed using 1.0 g of dried turmeric methanol extract dissolved in 3 mL of upper and 3 mL of lower phases. The mobile phase was the lighter, upper layer of the solvent system and the stationary phase was the heavier, lower layer. The fractions containing pure curcuminoids were combined together, evaporated to dryness on a rotary evaporator at 45 °C, analyzed for their purity by LC-ESI-Q-TOF-MS, and subjected to the antimicrobial assay.

## Figures and Tables

**Table 1:** LC-ESI-Q-TOF-MS accurate mass measurements of phenolics and terpenes identified in the MeOH and diethyl ether extracts of *Curcuma longa* tubers.

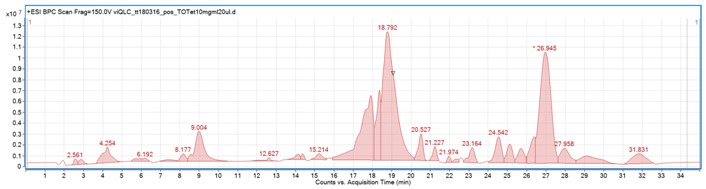							
**The total ion chromatogram of diethyl ether extract from turmeric tuber**							
**No.**	**Compound**	**Molecular Formula**	**Experimental Mass [M+H]^+^**	**Calculated Mass [M+H]^+^**	**Mass Error (ppm)**	**DBE**	**MS/MS Fragments**
1	CU	C_21_H_20_O_6_	369.1336	369.1333	−0.91	12	177, 145
2	DMCU	C_20_H_18_O_5_	339.1233	339.1227	−1.77	12	177, 147
3	BDMCU	C_19_H_16_O_4_	309.1122	309.1121	−0.21	12	147, 119
4	TUR	C_15_H_20_O	217.1586	217.1587	0.42	12	119, 91
5	Dihydro-BDMCU	C_19_H_18_O_4_	311.1286	311.1278	−2.63	11	225, 147
6	Dihydro-CU	C_21_H_22_O_6_	371.1468	371.1489	5.71	11	245, 177, 147
7	Dihydro-DMCU	C_20_H_20_O_5_	341.1377	341.1384	1.91	11	177, 147
8	Tetrahydro-BDMCU	C_19_H_20_O_4_	313.1286	313.1434	3.0	10	-

(DBE—double bond equivalent values; CU—curcumin; DMCU—dimethoxycurcumin; BDMCU—bisdemethoxycurcumin).

**Table 2:** Evaluation of the antimicrobial activity of four identified compounds against all tested Gram-positive bacteria strains, in relation to the isolated curcumin studied in a microdilution assay. The MIQ values are given in µg.

Strains of Bacteria	MIQ (µg)			
	CU	DMCU	BDMCU	TUR
*Staphylococcus aureus* ATCC 25923	4.42	0.91	0.57	-
*Staphylococcus aureus* ATCC 6538	0.55	0.23	0.28	-
*Staphylococcus aureus* ATCC 43300	8.84	1.83	0.57	0.23
*Staphylococcus epidermidis* ATCC 12228	0.55	0.23	0.14	1.80
*Bacillus subtilis* ATCC 6633	4.42	0.46	0.28	1.83
*Bacillus cereus* ATCC 10876	<0.28	<0.11	<0.14	-

**Table 3:** Determination of curcumin antibacterial activity in relation to vancomycin, by a standard microdilution assay, measured in µg.

	MIQ (µg)	
Strains of Bacteria	CU	Vancomycin
*Staphylococcus aureus* ATCC 25923	**25**	0.098
*Staphylococcus aureus* ATCC 6538	**6.25**	0.049
*Staphylococcus aureus* ATCC 43300	**50**	0.049
*Staphylococcus epidermidis* ATCC 12228	**6.25**	0.098
*Bacillus subtilis* ATCC 6633	**25**	0.024
*Bacillus cereus* ATCC 10876	**25**	0.098

**Table 4:** Determination of vancomycin equivalents for curcumin determined in the bioautographic and microdilution tests.

Strains of Bacteria	Calibration Curve Equation (Vancomycin)	Vancomycin Equivalents (TLC Bioutography)		Vancomycin Equivalents (Microdilution Assay)
		CU (µg ± SD)	RSD (%)	CU [µg]
*Staphylococcus aureus* ATCC 25923	y = 9731ln(x) − 12434	206 ± 12	5.8	255
*Staphylococcus aureus* ATCC 6538	y = 17492ln(x) − 7990.8	142 ± 10	6.9	127
*Staphylococcus aureus* ATCC 43300	y = 1381.9ln(x) + 5925.8	1310 ± 69	5.3	1020
*Staphylococcus epidermidis* ATCC 12228	y = 1340.3ln(x) + 128.79	49 ± 4	8.7	64
*Bacillus subtilis* ATCC 6633	y = 13889ln(x) + 22510	1328 ± 56	4.2	1041
*Bacillus cereus* ATCC 10876	y = 629.12ln(x) + 10915	202 ± 13	6.5	255

## Supplementary Materials

The following are available online at http://www.mdpi.com/1422-0067/20/4/898/s1: Figure S1 Purity of curcumin isolated by the centrifugal partition chromatography, Figure S2 EIC chromatograms of identified compounds present in the studied extracts of turmeric, Figure S3. Fragmentation patterns of curcuminoids isolated by centrifugal partition chromatography recorded in the CID collision energy of 20 V, Table S1. The identification of active zones on the TLC chromatograms by a TLC-MS interface coupled with a mass spectrometer.
