# Supplementary material for: Antimicrobial Potential of Single Metabolites of Curcuma longa Assessed in the Total Extract by Thin-Layer Chromatography-Based Bioautography and Image Analysis
Source: Int J Mol Sci. 2019 Feb 19;20(4):898. doi: 10.3390/ijms20040898 (PMC6412823; doi:10.3390/ijms20040898)
Supplement: Supplementary file 1 [file ijms-20-00898-s001.pdf]

SUPPLEMENTARY MATERIAL

Antimicrobial potential of single metabolites of *Curcuma longa* assessed in the total extract by thin-layer chromatography-based bioautography and image analysis

Lidia Czernicka, Agnieszka Grzegorczyk, Zbigniew Marzec, Beata Antosiewicz, Anna Malm, Wirginia Kukula-Koch*

Figure S1. Purity of curcumin isolated by the hydrostatic counter-current chromatography in the HPLC chromatogram recorded at 425 nm

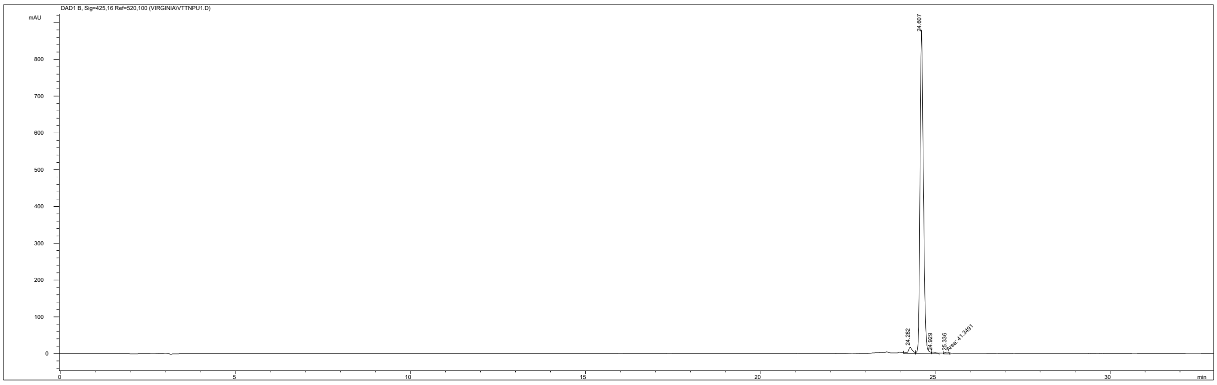

Figure S2. EIC chromatograms of identified compounds present in the studied extracts of turmeric

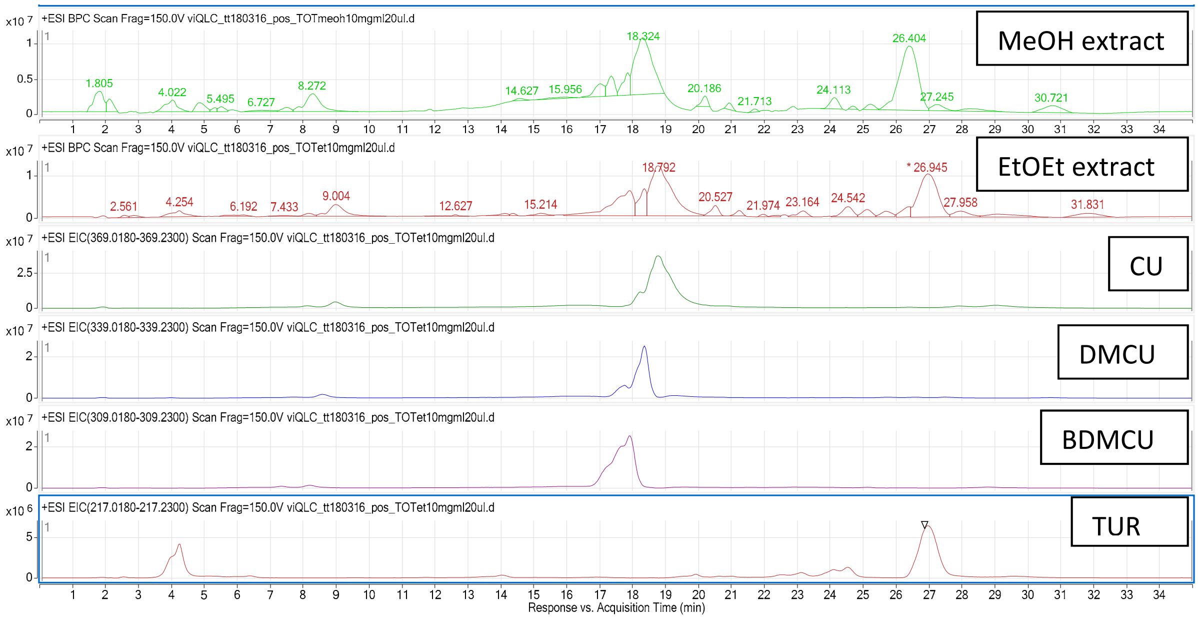

Figure S3. Fragmentation patterns of curcuminoids isolated by counter-current chromatography recorded in the CID collision energy of 20 V

Curcumin

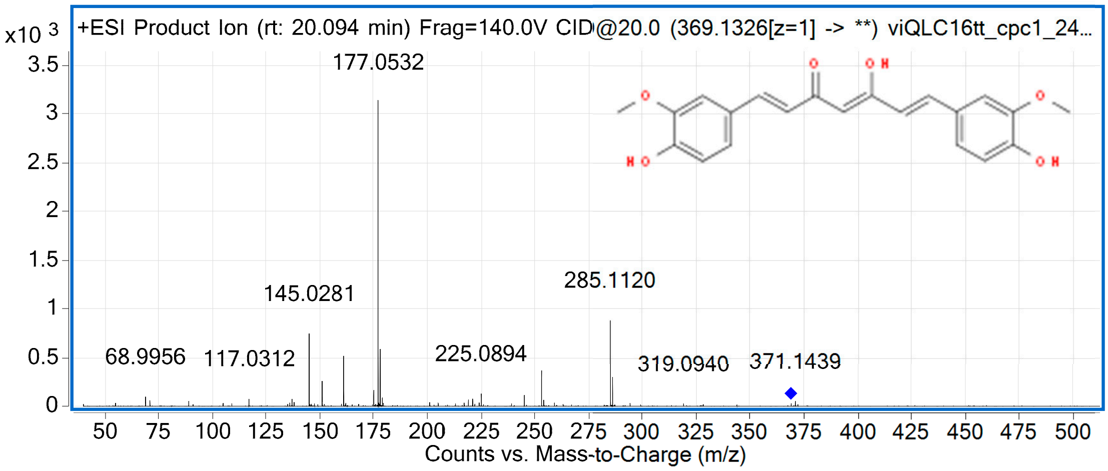

Demethoxycurcumin

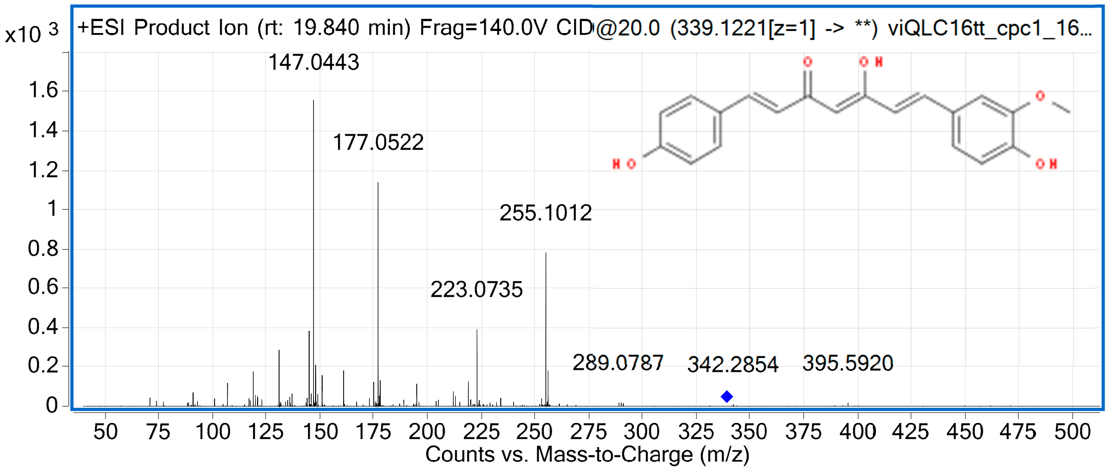

Bisdemethoxycurcumin

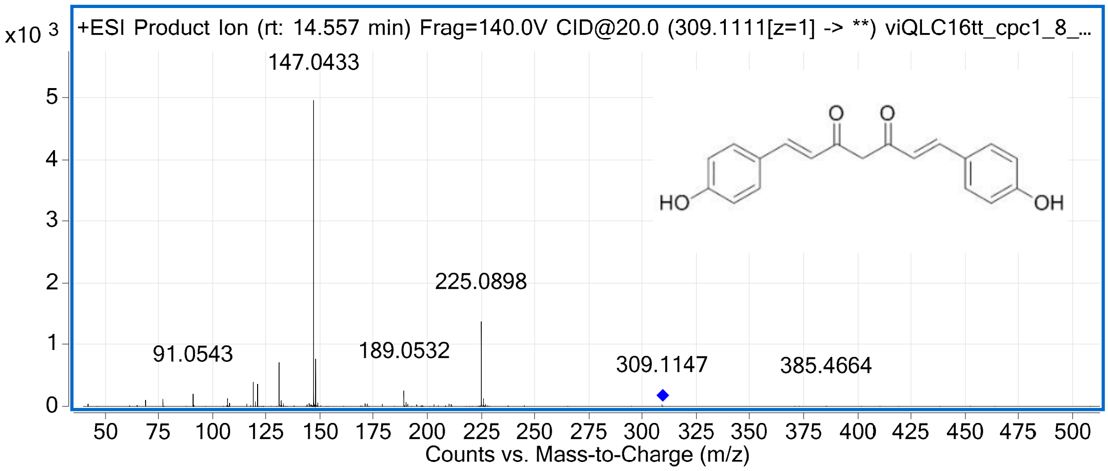

Table S1. The identification of active zones on the TLC chromatograms by a TLC-MS interface coupled with a mass spectrometer.

**Curcumin**<br>(CU)<br>[M+H]$^+$=369	**Demethoxycurcumin**<br>(DMCU)<br>[M+H]$^+$=339	**Bisdemethoxycurcumin**<br>(BDMCU)<br>[M+H]$^+$==309	**ar-Turmerone**<br>(TUR)<br>[M+H]$^+$=217
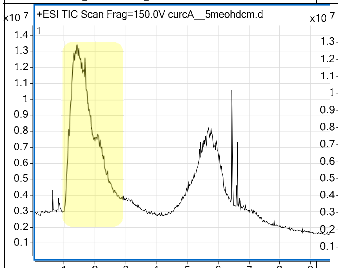	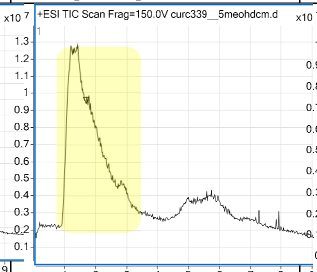	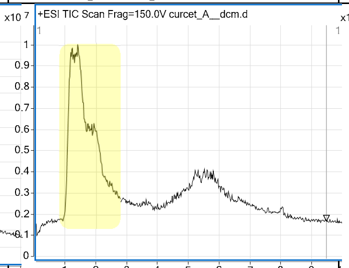	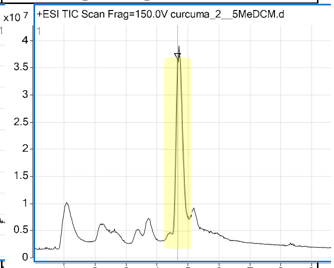
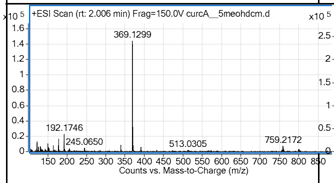	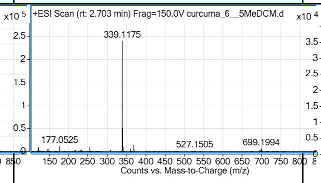	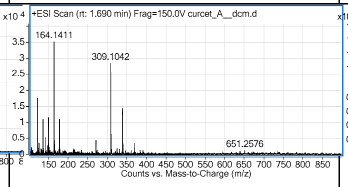	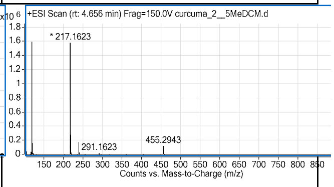
